# FOXD3 confers chemo-sensitivity in ovarian cancer through a miR-335/DAAM1/myosin II axis-dependent mechanism

**DOI:** 10.1186/s13048-022-01077-y

**Published:** 2023-01-10

**Authors:** Shufen Wang, Yan Ma, Yi Hu, Xia Zhao, Yilin Li, Shuming Ouyang, Guifang Luo

**Affiliations:** 1 The First Affiliated Hospital, Department of Gynecology, Hengyang Medical School, University of South China, Hengyang, 421001 Hunan China; 2 The First Affiliated Hospital, Reproductive Medicine Center, Hengyang Medical School, University of South China, Hengyang, 421001 Hunan China

**Keywords:** FOXD3, microRNA-335, DAAM1, Myosin II, Ovarian cancer, Chemoresistance

## Abstract

**Background:**

Chemotherapy is among the most common treatment methods for ovarian cancer (OC). However, chemoresistance limits the effectiveness of chemotherapy and leads to treatment failure. We herein investigate the biological effect of forkhead box D3 (FOXD3) in the chemoresistance of OC cells.

**Methods:**

Expression of FOXD3, miR-335 and disheveled-associated activator of morphogenesis 1 (DAAM1) was detected in OC cells and tissues. The regulatory network of FOXD3/miR-335/DAAM1 was validated by dual-luciferase reporter and ChIP assays *in vitro*. After ectopic expression and depletion experiments in carboplatin/paclitaxel (CP)-resistant (A2780CP) or sensitive (A2780S) OC cells, cell viability, colony formation and apoptosis were tested by CCK-8 assay, colony formation assay and flow cytometry respectively. Effects of FOXD3 on the chemoresistance of OC cells *in vivo* were evaluated in OC xenografts in nude mice.

**Results:**

Overexpression of FOXD3 impaired the proliferation and chemoresistance of OC cells, which was related to the promotion of the miR-335 expression. Functionally, DAAM1 was a putative target of miR-335. Silencing of DAAM1 was responsible for the inhibition of myosin II activation, consequently leading to suppressed OC cell proliferation and chemoresistance. *In vivo* results further showed that FOXD3 weakened the chemoresistance of OC cells to CP.

**Conclusion:**

Taken together, we unveil a novel FOXD3/miR-335/DAAM1/myosin II axis that regulates the chemoresistance of OC both *in vitro* and *in vivo*.

**Supplementary Information:**

The online version contains supplementary material available at 10.1186/s13048-022-01077-y.

## Background

Ovarian cancer (OC) is the third commonly occurring gynaecological cancer and the most lethal malignancy worldwide, associated with significant mortality [1]. Early age at menarche, late menopause, nulliparity and low parity, use of hormone replacement therapy and oral contraceptive use are well-established risk factors for OC [2]. Patients with OC often have a poor survival rate mainly due to the resistance to the chemotherapies, and drug resistance is a complex event that involves numerous genes and interactions between pathways [3]. Thus, identifying and understanding the underpinning molecular mechanisms relevant to chemoresistance are crucial for the management of treatment and development of novel and effective drug targets.

Forkhead box D3 (FOXD3) is a member of the forkhead box transcription factor family and often functions as a transcriptional repressor in the tumorigenesis of several types of cancers [4, 5]. FOXD3 could act as a tumor suppressor to impair cell viability and colony formation of OC A2780 cells [6]. The inhibiting effect of FOXD3 on the drug resistance has also been extensively reported in multiple cancers, including nasopharyngeal carcinoma and lung cancer [7, 8]. However, its potential effect on the OC chemoresistance has been rarely reported. microRNAs (miRNAs or miRs) have been highlighted to be a potential biomarker to predict the response of malignant tumor cells to chemotherapy owing to their roles in overcoming resistance or strengthening the sensitivity of tumor cells to chemotherapeutic agents [9]. A previously published report revealed that miR-335 was downregulated in drug-resistant OC cell lines and linked to the development of chemoresistance, representing a prognostic tool to monitor the chemotherapeutic outcomes [10].

Interestingly, miR-335 has been documented to post-transcriptionally target disheveled-associated activator of morphogenesis 1 (DAAM1) [11]. DAAM1 is an actin-associated regulatory factor and its overexpression results in promotion of OC cell migration and invasion [12]. Moreover, DAAM1 can activate Rho-ROCK1/2-myosin II and thus plays pivotal roles in modulating tumor-initiating potential and local invasion as well as distant metastasis [13]. Drug-resistant cancer cells are known to be mechanically heterogeneous, with subtypes of resistant cells exhibiting enhanced stiffness relative to their drug-sensitive cells, and myosin II has been involved in the mechanical alteration of drug-resistant cancer cells [14]. The above discussion revealed a possible network among FOXD3, miR-335, DAAM1, and myosin II in the OC chemoresistance development. Herein, our study was conducted in a bid to reveal the specific mechanism of FOXD3/miR-335/DAAM1/myosin II axis in OC.

## Results

### FOXD3 overexpression reduces the chemoresistance of OC cells

**Fig. 1:**
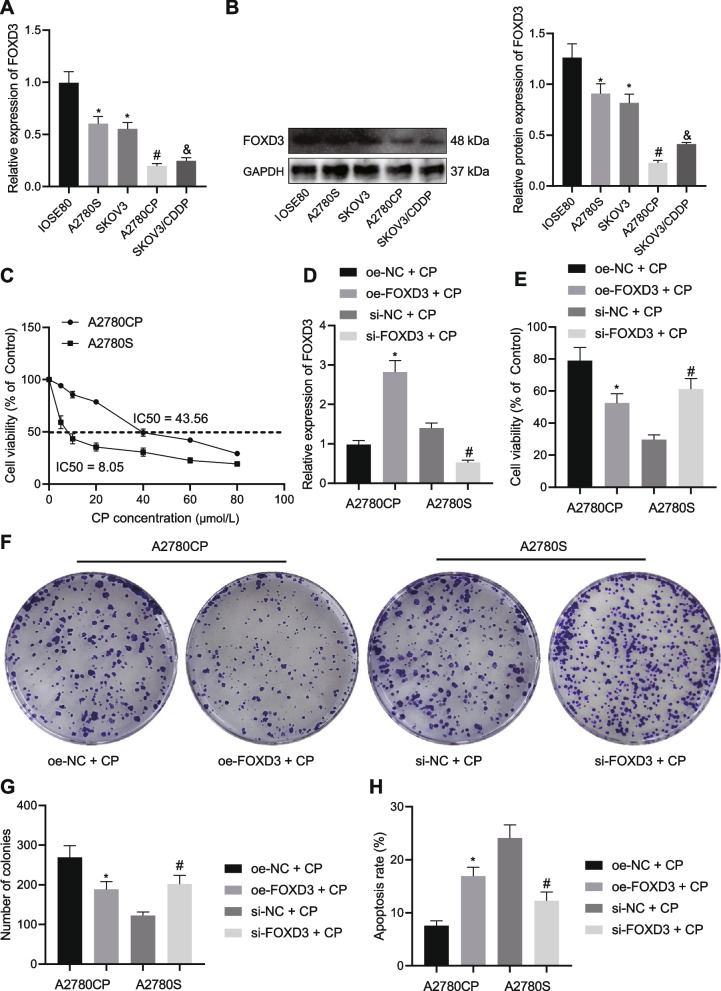
FOXD3 attenuates the chemoresistance of OC cells. **A** mRNA expression of FOXD3 determined by RT-qPCR in A2780S, SKOV3, IOSE80, A2780CP, and SKOV3/CDDP cell lines. **B** Western blot analysis of FOXD3 protein in A2780S, SKOV3, IOSE80, A2780CP, and SKOV3/CDDP cell lines. **C** IC_50_ values of A2780S and A2780CP cells treated with CP at different concentration measured by CCK-8 assay. A2780CP and A2780S cells were treated with CP and then transfected with oe-FOXD3 and si-FOXD3, respectively. **D**, mRNA expression of FOXD3 determined by RT-qPCR in A2780S and A2780CP cells. **E** Viability of CP-treated A2780S and A2780CP cells measured by CCK-8 assay. **F** Colony formation of CP-treated A2780S and A2780CP cells measured by colony formation assay. **G** Quantitative analysis of panel (**F**). **H** Flow cytometric analysis of the apoptosis of CP-treated A2780S and A2780CP cells. * *p* < 0.05, compared with IOSE80 cells or oe-NC + CP-treated A2780CP cells; # *p* < 0.05, compared with A2780S cells or si-NC + CP-treated A2780S cells; & *p* < 0.05, compared with SKOV3 cells. The experiment was conducted three times independently

Initially, RT-qPCR and Western blot data revealed lower mRNA and protein levels of FOXD3 in OC A2780S and SKOV3 cell lines than IOSE80 cell line. Meanwhile, FOXD3 was consistently downregulated in CP-resistant cell lines A2780CP and SKOV3/CDDP relative to A2780S and SKOV3 cell lines (Fig. 1A, B). The A2780CP and the sensitive cell line A2780S showing the lowest FOXD3 expression were used for follow-up experiments. CCK-8 data displayed that the IC50 of A2780S cells treated with CP for 48 h was 8.05 μmol/L, while the IC50 of A2780CP cells treated with CP was 43.56 μmol/L (Fig. 1C). As shown in Fig. 1D, FOXD3 expression was upregulated in A2780CP cells treated with oe-FOXD3 while it was reduced in A2780S cells treated with si-FOXD3. Accordingly, A2780CP cell viability and colony formation were attenuated in response to FOXD3 overexpression whereas a contrary trend was noted in the A2780S cells when FOXD3 was silenced (Fig. 1E-G). Furthermore, flow cytometric data presented that the apoptosis of A2780CP cells was increased following FOXD3 overexpression while that of A2780S cells was decreased following FOXD3 silencing (Fig. 1H). Altogether, FOXD3 boosted the apoptosis, inhibit the proliferation and attenuated the chemoresistance of OC cells.

### FOXD3 upregulates miR-335 which suppresses the chemoresistance of OC cells

**Fig. 2:**
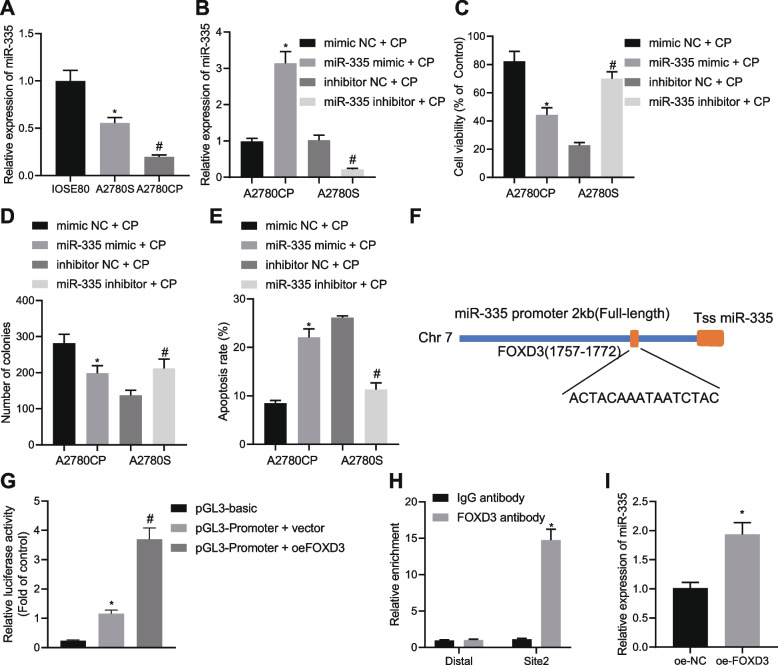
FOXD3 increases the expression of miR-335 and this increase restrains the chemoresistance of OC cells. **A** miR-335 expression determined by RT-qPCR in A2780S, IOSE80 and A2780CP cell lines. **B** miR-335 expression determined by RT-qPCR in miR-335 mimic + CP-treated A2780CP cells and miR-335 inhibitor + CP-treated A2780S cells. **C** Viability of miR-335 mimic + CP-treated A2780CP cells and miR-335 inhibitor + CP-treated A2780S cells measured by CCK-8 assay. **D** Colony formation of miR-335 mimic + CP-treated A2780CP cells and miR-335 inhibitor + CP-treated A2780S cells measured by colony formation assay. **E** Flow cytometric analysis of the apoptosis of miR-335 mimic + CP-treated A2780CP cells and miR-335 inhibitor + CP-treated A2780S cells. **F** The binding site between the promoter of miR-335 and FOXD3 predicted through the JASPAR website. **G** Binding of FOXD3 to the promoter of miR-335 determined by dual-luciferase reporter assay. **H** Enrichment of FOXD3 in the promoter region of miR-335 determined by ChIP assay. **I** miR-335 expression determined by RT-qPCR in oe-FOXD3-treated A2780CP cells. * *p* < 0.05, compared with IOSE80 cells, mimic NC + CP-treated A2780CP cells, pGL3-basic, IgG antibody or oe-NC-treated A2780CP cells; # *p* < 0.05, compared with A2780S cells, inhibitor NC + CP-treated A2780S cells or the pGL3-promoter vector. The experiment was conducted three times independently

We next aimed to analyze the downstream mechanism of FOXD3 in the chemoresistance of OC cells. RT-qPCR data exhibited lower miR-335 expression in A2780S and A2780CP cells than in IOSE80 cells, and A2780CP cells had a more pronounced decline of miR-335 expression than the A2780S cells (Fig. 2A). Figure 2B illustrates elevated miR-335 expression in A2780CP cells treated with oe-miR-335 and decreased miR-335 expression in A2780S cells treated with miR-335 inhibitor. Moreover, a decline of A2780CP cell viability and colony formation was induced following miR-335 overexpression whereas a contrary trend was noted in the A2780S cells in the absence of miR-335 (Fig. 2C, D). Meanwhile, A2780CP cell apoptosis was augmented upon miR-335 overexpression, the effect of which was abolished by miR-335 inhibition in A2780S cells (Fig. 2E).

In addition, the JASPAR website predicted a binding site between miR-335 and FOXD3 at the 2 KB promoter upstream of miR-335 (site: 1757–1772) with a sequence (ACTACAAATAATCTAC) (Fig. 2F). The results of dual-luciferase reporter assay confirmed that FOXD3 could target miR-335 and promote its expression (Fig. 2G). ChIP data also validated a significant enrichment of FOXD3 in the promoter region of miR-335 (Fig. 2H). Furthermore, miR-335 expression was experimentally determined to be upregulated in oe-FOXD3-treated A2780CP cells (Fig. 2I). The aforementioned data supported that FOXD3 upregulated miR-335 and this upregulation could reduce the chemoresistance of OC cells.

### DAAM1 is a target gene of miR-335

**Fig. 3:**
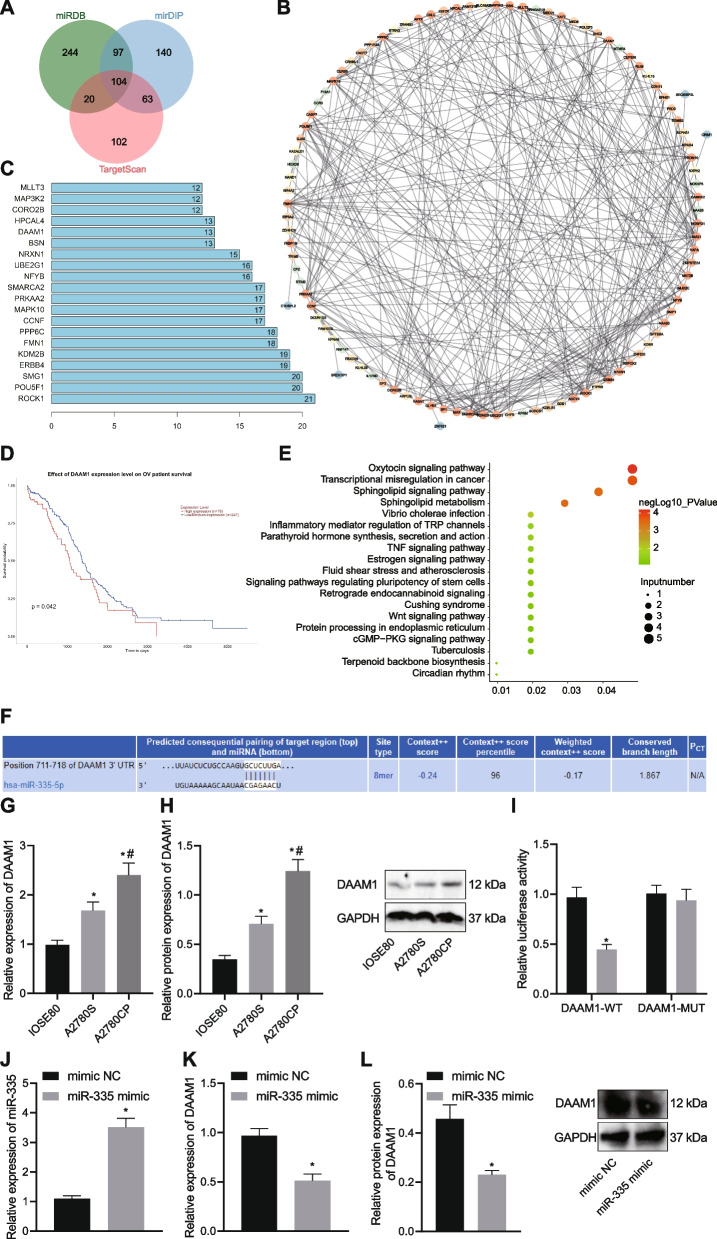
miR-335 targets DAAM1 and inhibits its expression in OC cells. **A** Venn diagram analysis of downstream target genes of miR-355 predicted by the miRDB, mirDIP, and TargetScan databases. The three circles in the figure represent the prediction results of three databases, and the central represents the intersection of three groups of data. **B** Interaction analysis of the candidate miRNA target genes; each circle in the figure represents a gene, and the line between circles indicates interaction between two genes; the more interacted genes exist in a gene, the higher the degree value, and the higher the core degree in the interaction network. **C** Degree values of core genes. The abscissa represents the degree value, and the ordinate represents the gene name. **D** KM survival curve indicating the correlation between the expression of DAAM1 and the survival rate of patients with OC. Red indicates patients with high expression of DAAM1 and blue indicates those with low expression of DAAM1. **E** KEGG enrichment analysis of candidate genes. The abscissa represents GeneRatio, the ordinate represents the entry identifier, the color represents enrichment *p* value, and the size represents the number of enriched genes in the identifier. **F** miR-335 targeted binding sites in the 3’UTR of DAAM1 predicted by the TargetScan database. **G** DAAM1 mRNA expression determined by RT-qPCR in A2780S, IOSE80 and A2780CP cell lines. **H** Western blot analysis of DAAM1 protein in A2780S, IOSE80 and A2780CP cell lines. **I** Binding of miR-335 to DAAM1 confirmed by dual-luciferase reporter assay in HEK-293 T cells. **J** miR-335 expression determined by RT-qPCR in miR-335 mimic-transfected A2780CP cells. **K** DAAM1 expression determined by RT-qPCR in miR-335 mimic-transfected A2780CP cells. **L** Western blot analysis of DAAM1 protein in miR-335 mimic-transfected A2780CP cells. * *p* < 0.05, compared with IOSE80 cells, or mimic NC-transfected A2780CP cells; # *p* < 0.05, compared with A2780S cells. The experiment was conducted three times independently

To investigate the miR-355-related molecular mechanism in the chemoresistance of OC cells, downstream target genes of miR-355 were predicted with multiple databases and subjected to Venn diagram analysis, with the results showing 104 candidate targets in the intersection (Fig. 3A). Following gene interaction network construction (Fig. 3B) and core gene degree value calculation (Fig. 3C), 20 candidate genes including DAAM1 were found at the core. Further, the Kaplan–Meier (KM) survival curves based on the expression of these 20 genes in OC patients were retrieved using the bioinformatics tool UALCAN database [15] (Supplementary Fig. 1). Among them, only DAAM1 shared a close correlation with the survival of patients with OC (*p* = 0.042), and the patients with high DAAM1 expression had lower survival rate (Fig. 3D). Therefore, we selected DAAM1 to be the target gene of miR-335. As shown in Fig. 3E, the 20 candidate genes including DAAM1 were significantly enriched in multiple signaling pathways, of which the most important signaling pathways were oxytocin signaling pathway, transcriptional misregulation in cancer, sphingolipid signaling pathway, and sphingolipid metabolism.

Meanwhile, the TargetScan database yielded putative miR-335 binding sites in the 3’UTR of DAAM1 sequence at 711 to 718 bp (Fig. 3F). Additionally, RT-qPCR and Western blot data exhibited higher DAAM1 expression in A2780S and A2780CP cells than in IOSE80 cells, and A2780CP cells had more increased DAAM1 expression than the A2780S cells (Fig. 3G, H). The luciferase activity of DAAM1-WT was diminished upon miR-335 mimic transfection in HEK-293 T cells (Fig. 3I), but that of DAAM1-3’-UTR-MUT had no significant change, suggesting that miR-335 could directly target and bind to the 3’-UTR region of DAAM1. The results of RT-qPCR and Western blot analysis further confirmed the binding of miR-335 to DAAM1, as shown by decreased DAAM1 expression in the miR-335 mimic-treated A2780CP cells (Fig. 3J-L). Therefore, DAAM1 could be a target that was negatively regulated by miR-335 in OC.

### FOXD3 suppresses the growth and chemoresistance of OC cells via regulation of the miR-335/DAAM1 axis

**Fig. 4:**
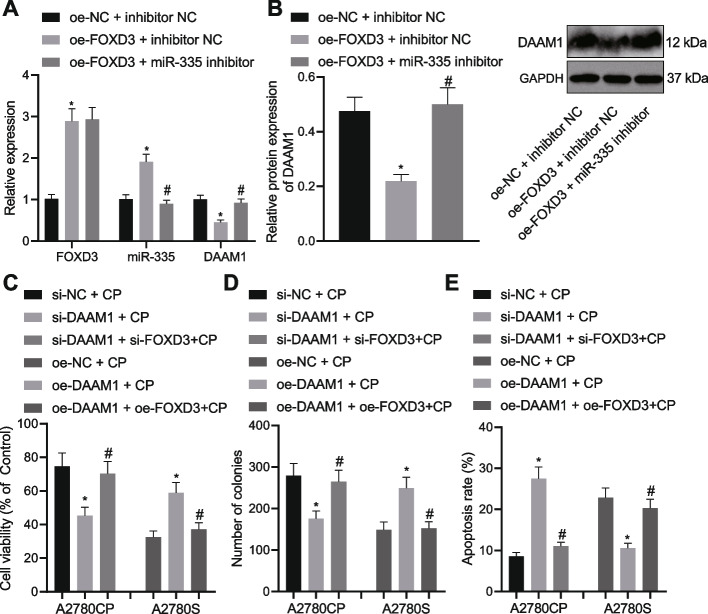
FOXD3 decelerates the growth and chemoresistance of OC cells via facilitation of the miR-335-mediated DAAM1 inhibition. **A** FOXD3, miR-335 and DAAM1 expression determined by RT-qPCR in A2780CP cells transfected with oe-FOXD3 or combined with miR-335 inhibitor. **B** Western blot analysis of DAAM1 protein in A2780CP cells transfected with oe-FOXD3 or combined with miR-335 inhibitor. CP-treated A2780CP cells were transfected with si-DAAM1 or combined with si-FOXD3, while CP-treated A2780S cells were transfected with oe-DAAM1 or combined with oe-FOXD3. **C** Viability of A2780S and A2780CP cells measured by CCK-8 assay. **D** Colony formation of A2780S and A2780CP cells measured by colony formation assay. **E** Flow cytometric analysis of the apoptosis of A2780S and A2780CP cells. * *p* < 0.05, compared with A2780CP cells transfected with oe-NC + inhibitor NC or treated with si-NC + CP or A2780S cells treated with oe-NC + CP; # *p* < 0.05, compared with A2780CP cells transfected with oe-FOXD3 + inhibitor NC or treated with si-DAAM1 + CP, or A2780S cells treated with oe-DAAM1 + CP. The experiment was conducted three times independently

Next, we sought to test whether FOXD3 inhibited the growth and chemoresistance of OC cells by upregulating miR-335 and inhibiting DAAM1. Our RNA and protein quantitation results demonstrated elevations in the FOXD3 mRNA expression and miR-335 expression in A2780CP cells overexpressing FOXD3 while the DAAM1 mRNA and protein expression was downregulated. A contrasting trend was evident following dual transfection with oe-FOXD3 and miR-335 inhibitor (Fig. 4A, B). This result suggested that FOXD3 could inhibit the expression of DAAM1 by upregulating miR-335.

Additionally, A2780CP cell viability and colony formation were weakened and their apoptosis was accelerated following DAAM1 silencing whereas simultaneous silencing of DAAM1 and FOXD3 resulted in opposite results. Besides, oe-DAAM1-treated A2780S cells showed enhanced viability and colony formation and repressed apoptosis but contrary changes were observed following further FOXD3 overexpression (Fig. 4C-E). Taken together, the inhibiting effect of FOXD3 on the growth and chemoresistance of OC cells was related to its regulation on the miR-335/DAAM1 axis.

### DAAM1 silencing represses myosin II activation in CP-resistant OC cells

**Fig. 5:**
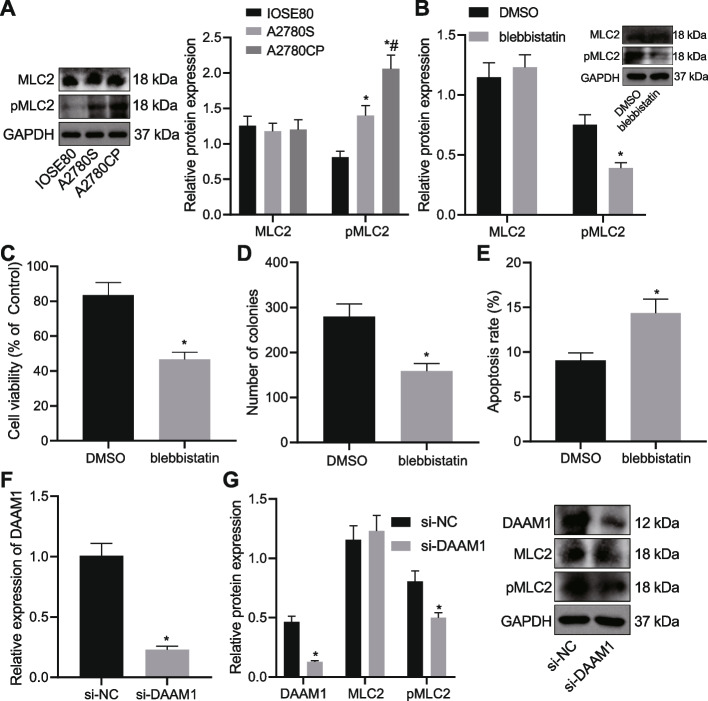
DAAM1 knockdown blunts myosin II activation in CP-resistant OC cells. **A** MLC2 expression and the extent of MLC2 phosphorylation determined by Western blot analysis in A2780S, A2780CP and IOSE80 cells. **B** MLC2 expression and the extent of MLC2 phosphorylation determined by Western blot analysis in blebbistatin-treated A2780CP cells. **C** Viability of blebbistatin-treated A2780CP cells measured by CCK-8 assay. **D** Colony formation of blebbistatin-treated A2780CP cells measured by colony formation assay. **E** Flow cytometric analysis of the apoptosis of blebbistatin-treated A2780CP cells. **F** DAAM1 mRNA expression determined by RT-qPCR in A2780CP cells treated with si-DAAM1. **G** DAAM1 and MLC2 protein expression as well as the extent of MLC2 phosphorylation determined by Western blot analysis in A2780CP cells treated with si-DAAM1. * *p* < 0.05, compared with IOSE80 cells, or A2780CP cells treated with DMSO or si-NC; # *p* < 0.05, compared with A2780CP cells. The experiment was conducted three times independently

Western blot analysis suggested no alterations in the MLC2 expression in A2780S and A2780CP cells as compared to IOSE80 cells while the extent of MLC2 phosphorylation was increased, with A2780CP cells showing the higher extent (Fig. 5A). Treatment with blebbistatin led to decreased extent of MLC2 phosphorylation in A2780CP cells (Fig. 5B). We observed a reduction in the viability and colony formation of A2780CP cells and an enhancement in the A2780CP cell apoptosis in the presence of blebbistatin (Fig. 5C-E), indicating that inhibition of MLC2 can impede the proliferation while stimulating apoptosis of CP-resistant OC cells. We then silenced DAAM1 in A2780CP cells by manipulation with si-DAAM1 (Fig. 5F). Additional Western blot results displayed that treatment of si-DAAM1 decreased the DAAM1 expression and the extent of MLC2 phosphorylation (Fig. 5G). The above data indicated that DAAM1 knockdown could disrupt the myosin II activation in CP-resistant OC cells.

### The FOXD3/miR-335/DAAM1 axis represses the chemoresistance of OC cells by downregulating myosin II

**Fig. 6:**
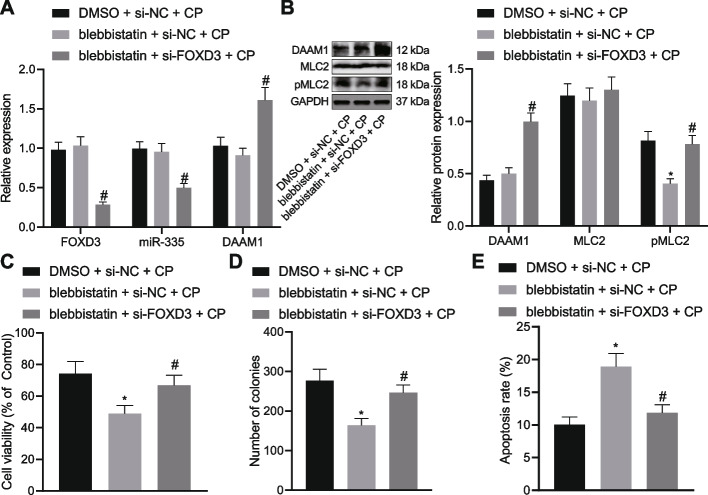
FOXD3/miR-335/DAAM1 affects chemoresistance of OC cells by regulating myosin II. CP-treated A2780CP cells were treated with blebbistatin or combined with si-FOXD3. **A** mRNA expression of FOXD3 and DAAM1 as well as miR-335 expression determined by RT-qPCR in A2780CP cells. **B** DAAM1 and MLC2 protein expression as well as the extent of MLC2 phosphorylation determined by Western blot analysis in A2780CP cells. **C** A2780CP cell viability measured by CCK-8 assay. **D** Colony formation of A2780CP cells measured by colony formation assay. **E** Flow cytometric analysis of A2780CP cell apoptosis. * *p* < 0.05, compared with A2780CP cells treated with DMSO + si-NC + CP; # *p* < 0.05, compared with A2780CP treated with blebbistatin + si-NC + CP. The experiment was conducted three times independently

Next, we shifted our attention to determine whether the FOXD3/miR-335/DAAM1 axis can reduce OC resistance by inhibiting myosin II activation. It was detected that the extent of MLC2 phosphorylation was reduced following treatment with blebbistatin while further treatment with si-FOXD3 downregulated the FOXD3 mRNA expression and miR-335 expression, and increased DAAM1 mRNA and protein expression and MLC2 phosphorylation level (Fig. 6A, B). These results indicated that silencing of FOXD3 promoted myosin II activation. In addition, A2780CP cells treated with blebbistatin presented with diminished viability and colony formation and augmented apoptosis. However, combined treatment with blebbistatin and si-FOXD3 resulted in stimulated A2780CP cell viability and colony formation and reduced apoptosis (Fig. 6C-E). In conclusion, FOXD3 silencing resulted in downregulation of miR-335 and upregulation of DAAM1 to activate myosin II and induce chemoresistance of OC cells.

### FOXD3 enhances the sensitivity of chemo-resistant OC cells to CP *in vivo*

**Fig. 7:**
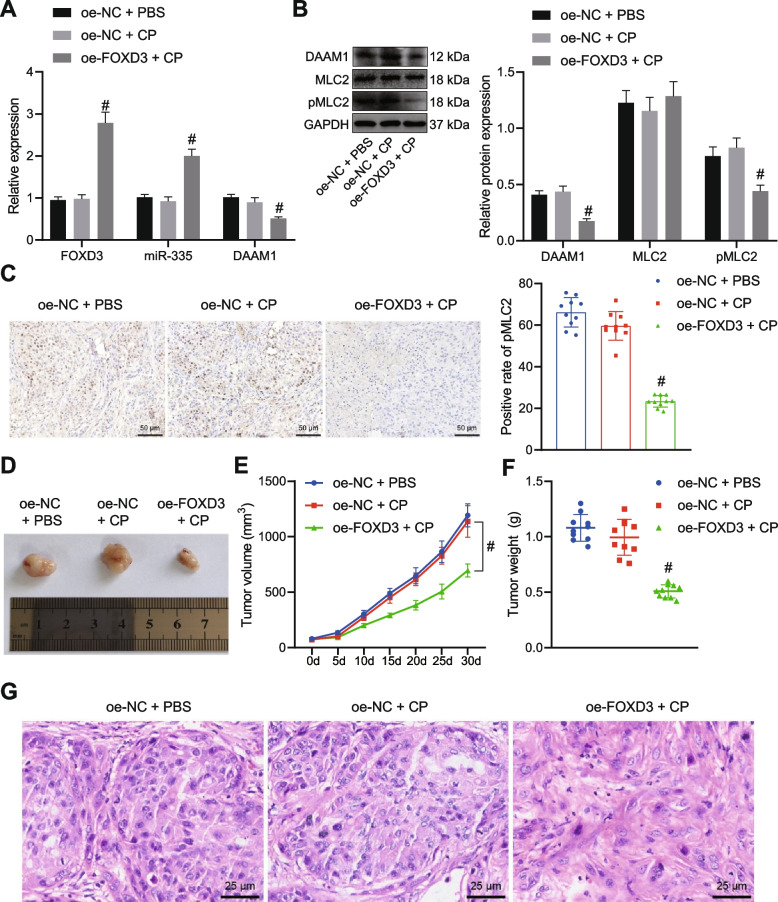
FOXD3 increases the sensitivity of OC cells to CP *in vivo*. Mice were treated with CP or combined with oe-FOXD3. **A** Expression of FOXD3, miR-335 and DAAM1 determined by RT-qPCR in mouse tumor tissues. **B** DAAM1 and MLC2 protein expression as well as the extent of MLC2 phosphorylation determined by Western blot analysis in mouse tumor tissues. **C** Positive expression of pMLC2 in mouse tumor tissues determined by Immunohistochemical staining (scale bar = 50 μm). D, Representative images showing xenografts in nude mice. **E** Tumor volume of mice measured every five days. **F** Tumor weight of mice. **G** HE staining of mouse tumor tissues (scale bar = 25 μm). # *p* < 0.05, compared with mice treated with oe-NC + CP. *N* = 10 for mice following each treatment

The aforementioned *in vitro* effect of FOXD3 on the chemoresistance of OC cells was further validated in nude mice. As depicted in Fig. 7A, B, FOXD3 mRNA and protein expression and miR-335 expression were upregulated while DAAM1 mRNA and protein expression along with extent of MLC2 phosphorylation was reduced in tumor tissues of mice co-treated with oe-FOXD3 and CP. Meanwhile, the positive pMLC2 expression was decreased in tumor tissues of CP-treated mice following overexpression of FOXD3 (Fig. 7C). Meanwhile, overexpression of FOXD3 reduced tumor volume and weight of CP-treated mice (Fig. 7D-F). HE staining analysis indicated obvious necrosis and inflammatory cell infiltration in tumor tissues of mice upon combined treatment with oe-FOXD3 and CP (Fig. 7G). Cumulatively, overexpression of FOXD3 could advance the sensitivity of OC cells to CP *in vivo*.

## Discussion

Chemoresistance often causes treatment failure and is the main obstacle for OC treatment during clinical therapy [16]. Development of new strategies for overcoming chemoresistance is thus the central goal in OC research. The findings gained from the present study uncovered the inhibiting property of FOXD3 in the malignant characteristics of OC cells and chemoresistance to CP via miR-335-mediated DAAM1 inhibition and the resultant myosin II inactivation.

Initial results indicated that overexpression of FOXD3 could promote the apoptosis while inhibiting the proliferation and chemoresistance of OC cells. FOXD3 is poorly expressed in OC tissues while its overexpression weakens OC cell proliferating and migrating ability and increases cell apoptosis while restraining tumor growth *in vivo* [17]. FOXD3 expression is downregulated in CP-resistant nasopharyngeal carcinoma cells and it can promote the expression of miR-26b which sensitizes nasopharyngeal carcinoma cells to CP [7]. In addition, FOXD3 can repress the drug resistance of lung cancer cells *in vitro* and *in vivo* [8], which is in line with our results that overexpression of FOXD3 could attenuate the chemoresistance of OC cells to CP *in vivo*.

FOXD3 was previously unveiled to positively regulate the expression of miRNAs, such as miR-214 and miR-26b [7, 18]. This study first demonstrated that FOXD3 upregulated miR-335 by enriching in the promoter region of miR-335. miR-335-5p is weakly expressed in CP-resistant A2780 cells while its overexpression can reduce cell viability and enhance apoptosis and the sensitivity of OC cells to CP by suppressing its target gene BCL2L2 [19]. Therefore, the suppressing impact of FOXD3 on the growth of OC cells and chemoresistance to CP was associated with miR-335 upregulation.

Further investigation revealed that DAAM1 was a downstream target inhibited by miR-335. Consistently, miR-335-5p can directly target the 3’UTR of DAAM1 and consequently result in the repression of the DAAM1 expression [20]. Meanwhile, increased expression of DAAM1 is closely associated with the distant metastasis in OC and siRNA-induced silencing of DAAM1 impairs the migration and invasion potentials of OC cells [12]. Thus, the miR-335-mediated DAAM1 suppression may serve as therapeutic strategies for the management of OC chemoresistance. Further mechanistic findings suggested that DAAM1 silencing repressed myosin II activation in drug-resistant OC cells. Consistently, DAAM1 is capable of activating Rho-ROCK1/myosin II, and the Wnt11/5B-FZD7-DAAM1 axis could upregulate MLC2 phosphorylation and myosin II activity by activating RhoA-ROCK1/2 signal [13]. Therefore, we speculate that silencing of DAAM1 in A2780CP cells may reduce MLC2 phosphorylation by inhibiting RhoA-ROCK1/2 signal, which warrants further validation. Meanwhile, myosin II is overexpressed in drug-resistant OC cells and essential for the migration of drug-resistant cells via modulating nuclear deformability and protease activity [21], suggesting the potential of myosin II activation to enhance the drug resistance of OC cells. Additionally, the restored activity of myosin II in therapy-resistant melanoma cells can increase cell survival while its ablation specifically kills resistant cells through intrinsic lethal reactive oxygen species and unresolved DNA damage [22]. Overall, the aforementioned results provided evidence that FOXD3 facilitated the miR-335-mediated DAAM1 inhibition and disrupted myosin II activity, thus impeding the growth and chemoresistance of OC cells. However, owing to the limited supportive data for the interaction between FOXD3, myosin II and DAAM1, miR-335 and myosin II, our subsequent endeavors are necessary for validating the potential of this newly discovered mechanism.

## Conclusion

**Fig. 8:**
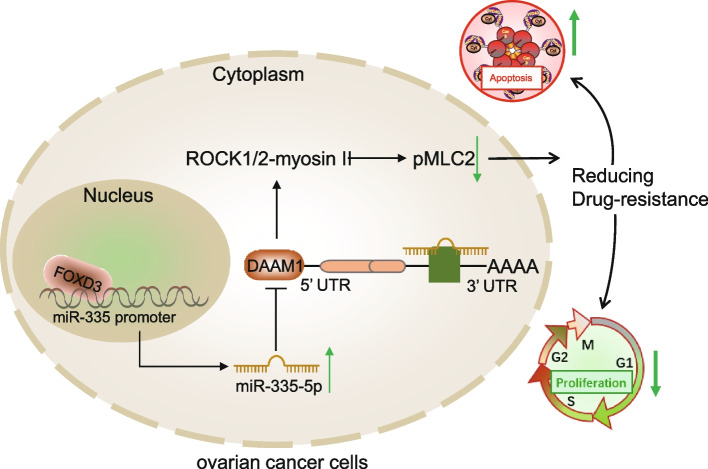
Schematic diagram of the mechanism by which FOXD3/miR-335/DAAM1 affects the chemoresistance of OC cells. FOXD3 can inhibit the expression of DAAM1 by promoting miR-335 and reduce the activity of myosin II, thus inhibiting the proliferation, augmenting apoptosis and reducing the chemoresistance of OC cells

To sum up, FOXD3 directly enhanced the miR-335 expression and sequentially downregulated the DAAM1 expression, which inhibited the activity of myosin II, and alleviated the chemoresistance of OC cells to CP (Fig. 8). This might well aid intervention strategies of OC in the future. Nevertheless, experiments performed in the present study were exclusively *in vitro* and *in vivo*, which limited the further clinical significance of our results. Further studies are needed to expand our understanding of the anti-chemoresistance function of FOXD3 in the specimens from OC-diagnosed patients, in order to potentiate its clinical applications.

## Materials and methods

### Ethics statement

The study was approved by the Animal Ethics Committee of The First Affiliated Hospital of University of South China and strictly conformed to the Guidelines for the Care and Use of Laboratory Animals, with minimal suffering ensured.

### Microarray-based gene expression profiling

The target genes of miR-335 was predicted using the miRDB, mirDIP, and TargetScan databases, followed by intersection analysis. The STRING database was used for interaction analysis of miR-335 target genes and interaction networks were constructed with cytoscape v3.7.1, with the degree value of core genes calculated. Finally, KEGG enrichment analyses were followed with the assistance of the KOBAS3.0 database.

### Cell culture and treatment

Normal human ovarian epithelial cell line IOSE80, human OC cell lines A2780S and SKOV3 were all purchased from Meisen (Hangzhou, China). The corresponding CP-resistant OC cell lines A2780CP and SKOV3/CDDP were constructed by our laboratory. Sensitive parental A2780S cells and SKOV3 cells were co-cultured with 10–80 μM CP (Sigma-Aldrich, St. Louis, MO) for 48 h, during which the concentration of CP was gradually increased [23, 24]. After resuscitation, these cells were cultured in DMEM (Gibco, Carlsbad, CA) containing 10% FBS (Gibco), 100 U/mL penicillin and 100 μg/mL streptomycin in a 5% CO_2_ incubator (BB15, Thermo Fisher Scientific Inc., Waltham, MA) at 37℃. The medium was renewed every 24 h, and cells were treated with 0.25% trypsin (Hyclone Laboratories, Logan, UT) every 72 h and sub-cultured. Before the experiment, A2780CP cells were treated with 9 μg/mL CP (Sigma-Aldrich) for one week to maintain drug resistance.

The log-phase cells were trypsinized, seeded into 6-well plates, and cultured for 24 h. Upon reaching approximately 60% cell confluence, transfection was conducted as per the Lipofectamine 3000 transfection reagent (Thermo Fisher Scientific) with the following plasmids: oe-NC, oe-FOXD3, si-NC, si-FOXD3, mimic NC, miR-335 mimic, inhibitor NC, miR-335 inhibitor, oe-NC + inhibitor NC, oe-FOXD3 + inhibitor NC, oe-FOXD3 + miR-335 inhibitor, si-DAAM1, si-DAAM1 + si-FOXD3, oe-DAAM1 and oe-DAAM1 + oe-FOXD3. These aforementioned plasmids were synthesized by GenePharma (Shanghai, China). The medium was replaced with complete medium after 6 – 8 h of culture, followed by continued culture till 48 h. The cells were collected and used for subsequent experimentation. Then, 10 μM Myosin II inhibitor blebbistatin (Cat#120,491, Abcam Inc., Cambridge, UK) was prepared by dissolving in DMSO and subsequently added to the cells 4 h after cell seeding to facilitate adherence of the cell matrix [21].

### CCK-8 assay

Cells were seeded into 96-well plates with 1 × 10^4^ cells per well and cultured for 24 h. When IC50 was detected, OC cells were incubated with 0, 5, 10, 20, 40 and 80 μmol/L CP (Sigma-Aldrich) for 48 h. CP was soluble in water, and the mother solution was thus prepared with distilled water before the experiment and cryopreserved at—20℃ for subsequent cell viability test. The concentration of CP was 25 μg/mL and the cells were incubated with CP for 48 h. Each well was subsequently supplemented with 10 µL of CCK-8 solution (Sigma-Aldrich) for 1-h incubation in a 37℃ incubator for 1 h. The OD value of each well was measured using an Epoch microplate spectrophotometer (Omega Bio-tek Inc, Norcross, GA) at 450 nm.

### Colony formation assay

Log-phase cells were digested with 0.25% trypsin and resuspended. The cells in 6-well plates (1 × 10^3^ cells/well) were cultured to form cell colonies within two weeks. During the transfection experiment, the medium was renewed every 2 days following 12 h of transfection. On day 6, the cells were transfected again. After another 6 days, the cells were fixed in 4% paraformaldehyde and dyed by 0.5% crystal violet (0.5% w/v, Solarbio, Beijing, China) for 15 min. Cell colonies (over 50 cells) were counted under a stereomicroscope.

### Flow cytometry

Annexin V-FITC/PI double staining kit (70-AP101-100, Lanke Biotechnology Co., Ltd., Hangzhou, Zhejiang, China) was employed to examine cell apoptosis. The cells were detached with 0.25% trypsin without EDTA, centrifuged at 300 × g for 5 min, and resuspended in 500 µL of binding buffer. Next, 3 μL Annexin V-FITC and 2.5 μL PI were supplemented to the cells for 20-min incubation at 37℃ under dark conditions. A FACSCalibur flow cytometer (BD Bioscience, Franklin Lakes, NJ) was utilized for analysis.

### RNA extraction and quantification

Cellular total RNA and total RNA in tissues were isolated with TRIzol reagent (15596026, Invitrogen, Carlsbad, CA). Next, the extracted RNA was reversely transcribed into cDNA with PrimeScript™ RT reagent Kit (RR047A, Takara, Japan) or PolyA tailing kit (B532451, Sangon, Shanghai, China). RT-qPCR was then performed using Fast SYBR Green PCR Kit (Applied Biosystems, Foster City, CA) and the ABI PRISM 7300 RT-PCR system (Applied Biosystems). U6 was employed as an internal reference for miR-335 and GAPDH for the remaining genes (Supplementary Table 1). The fold changes were calculated by means of relative quantification (2^−△△Ct^ method).

### Western blot analysis

Cellular total protein and total protein in tissues were obtained using PMSF-containing RIPA lysis buffer (Beyotime Biotechnology Co., Shanghai, China) and then quantified using a BCA protein assay kit (Pierce, Rockford, IL). Afterwards, 50 μg protein following electrophoresis were transferred onto a PVDF membrane. The membrane blocked by 5% skim milk powder was then probed overnight at 4℃ with primary antibodies against FOXD3 (mouse, sc-517206, 1:500, Santa Cruz Biotechnology, Inc, Santa Cruz, CA), DAAM1 (mouse, sc-100942, 1:500, Santa Cruz Biotechnology), phosphorylated myosin light chain 2 (p-MLC2; Cat#3674S, 1:1000, Cell Signaling Technologies, Beverly, MA), MLC2 (Cat#3672, 1:1200, Cell Signaling Technologies) and GAPDH (mouse, sc-47724, 1:100–1:1000, Santa Cruz Biotechnology, serving as a loading control). HRP-conjugated secondary goat anti-mouse IgG (1:10,000, Boster Biological Technology Co., Ltd., Wuhan, Hubei, China) was supplemented for 1 h of re-probing. The immunocomplexes on the membrane were visualized using enhanced chemiluminescence reagent, and quantitative analysis of band intensities was done using Image J software, with the expression of GAPDH as the internal reference.

### Dual luciferase reporter assay

The sequence containing the targeted binding site between miR-335 and DAAM1 was obtained through TargetScan database. The DAAM1-3’UTR-WT or DAAM1-MUT sequence was synthesized and cloned into pmiR-RB-Report vector (RiboBio, Guangzhou, China). All the plasmids were extracted employing Omega plasmid small amount extraction kit (D1100-50 T, Solarbio), and the constructed recombinant plasmids were transformed and amplified. After the cell adherence, DAAM1-3'UTR-WT and DAAM1-3'UTR-MUT reporter plasmids were co-transfected with miR-335-5p mimic/NC mimic into HEK-293 T cells (2 × 10^5^ cells/well) in 6-well plates. After 48 h of culture, the cells were collected for luciferase activity detection.

The JASPAR website was adopted to acquire the putative binding sites of FOXD3 in miR-335-5p promoter. The 2 kb region upstream of miR-335-5p was amplified by PCR, and then the product was cloned into the vector. The binding of FOXD3 to the promoter of miR-335-5p was detected by transient transfection of pGL3 promoter/oe-FOXD3/pRL-TK, pGL3 promoter/vector/PRL-TK or pGL3-basic/pRL-TK into HEK293T cells. Following 48 h of transfection, the changes in luciferase activity were detected using a dual-luciferase assay detection kit (D0010, Solarbio) on a Glomax 20/20 luminometer (E5311, Shaanxi Zhongmei Biotechnology Co., Ltd., Shaanxi, China).

### ChIP

Cells were fixed with 1% formaldehyde for 10 min to produce DNA–protein cross-linking. Then, the cells lysate was subjected to ultrasonic treatment to generate 200—1000 bp chromatin fragments. An overnight incubation was followed at 4℃ with antibodies against FOXD3 (sc-517206, Santa Cruz Biotechnology) and IgG (serving as NC). The Pierce protein A/G beads (88,803, Thermo Fisher Scientific) were used to immunoprecipitate the DNA that could bind to FOXD3. After 5-min centrifugation at 12,000 × g and washing, the DNA–protein complex was incubated at 65℃ overnight to relieve cross-linking. Ultimately, the precipitated DNA was analyzed by RT-qPCR using Bio-Rad iQ SYBR green supermix.

### OC xenografts in nude mice

Thirty female BALB/c nude mice (aged 4–5 weeks old and weighing 18—22 g) were purchased from Shanghai SLAC Laboratory Animal Co., Ltd. (Shanghai, China). The mice were housed at 25—27℃ with 45—50% humidity for one week with a 12-h light/dark cycle. The mice were given ad libitum access to food and water but fasted for 12 h prior to subcutaneous administration. The mice were randomly grouped into 3 groups, 10 mice in each group: oe-NC + PBS, oe-NC + CP and oe-FOXD3 + CP. The A2780CP cells stably transfected with oe-NC and oe-FOXD3 were subcutaneously injected into the location 1–2 cm under the right armpit of mice at a density of 1 × 10^7^ cells/mouse (0.2 mL). When the tumor volume reached 80 mm^3^, CP (2 mg/kg) was injected into the tumor with a micro-syringe, once every 5 days. The weight of mice was weighed before each administration, and the tumor growth was monitored by measuring the tumor width (W) and length (L) with Vernier calipers, and calculated using the formula: tumor growth = (W^2^ × L)/2. After 30 days, the mice were euthanized and the tumor tissue was removed. The isolated tumors were photographed with a camera after weighting.

### HE staining

OC tissues were fixed with 4% paraformaldehyde, paraffin-embedded and sectioned. The sections were heated at 60℃ for 1 h, dewaxed with xylene, hydrated with gradient alcohol and stained with hematoxylin (Solarbio) for 2 min. Following tap water washing for 10 s, the sections were immersed in 1% hydrochloric acid–ethanol for 10 s, and colored with eosin (Solarbio) for 1 min. The sections were lastly subjected to observation with the XP-330 optical microscope (Shanghai Bingyu Optical Instrument Co., Ltd., Shanghai, China).

### Immunohistochemical staining

OC tissues collected from nude mice were fixed with 4% paraformaldehyde, paraffin-embedded and cut into 4-μm-thick sections. The sections were subjected to microwave-stimulated antigen retrieval in 0.1 M citric acid buffer (pH 6.0) for 10 min and then immunostained with primary antibody against MLC2 (phosphor-Ser 19) (11114, Signalway Antibody Co., College Park, MD, USA) at 4℃ overnight. Goat anti-rabbit (ab6721, Abcam) labeled by HRP was utilized as secondary antibody. Following that, 0.05% DAB containing 0.01% hydrogen peroxide (Beijing Bioss Biotechnology Co. Ltd., Beijing, China) was employed for color development. Thereafter, the sections were stained with hematoxylin for 5 min, soaked in 1% hydrochloric acid–ethanol for 4 s, and blued with tap water for 20 min. The cells with brownish-yellow nuclei were normal positive cells. Image-Pro Plus software (Version X; Media Cybernetics, Silver Springs, MD) was adopted to detect the average OD value of positive cells under a high power microscope, followed by quantitative analysis of protein expression: five high power microscope fields/section and 200 cells/field were observed.

### Statistical analysis

All data were analyzed using SPSS 22.0 statistical software (IBM Corp. Armonk, NY) Graphpad Prism 7.0 (Graphpad software, La Jolla, CA). The measurement data were described as mean ± standard deviation. Data between two groups were compared by unpaired *t*-test. Differences among multiple groups were statistically analyzed employing one-way ANOVA and Tukey’s multiple comparisons test. Data among multiple groups depending on time points were compared with repeated measures ANOVA with Tukey’s post hoc test. A value of *p* < 0.05 was statistically significant.

## Supplementary Information

**Additional file 1: Supplementary Fig. 1.** KM survival curves based on 19 candidate genes in OC. Red indicates patients with high gene expression and blue indicates those with low gene expression.

**Additional file 2: Supplementary Table 1.** Primer sequences for RT-qPCR.

## Data Availability

The datasets generated and/or analyzed during the current study are available from the corresponding author on reasonable request.

## Abbreviations

OC: Ovarian cancer
miRNAs/miRs: MicroRNAs
DAAM1: Disheveled-associated activator of morphogenesis 1
FOXD3: Forkhead box D3
RT-qPCR: Reverse transcription quantitative polymerase chain reaction
oe: Overexpression
si: Small interfering RNA
CP: Cis-platinum
KEGG: Kyoto encyclopedia of genes and genomes
DMEM: Dulbecco's modified Eagle's medium
FBS: Fetal bovine serum
NC: Negative control
DMSO: Dimethyl sulphoxide
OD: Optical density
FITC/PI: Fluorescein isothiocyanate/propidium iodide
EDTA: Ethylenediaminetetraacetate
cDNA: Complementary DNA
GAPDH: Glyceraldehyde-3-phosphate dehydrogenase
RIPA: Radioimmunoprecipitation assay
BCA: Bicinchoninic acid
PVDF: Polyvinylidene fluoride
HRP: Horseradish peroxidase
ChIP: Chromatin immunoprecipitation
ANOVA: Analysis of variance
