# Supplementary material for: FOXD3 confers chemo-sensitivity in ovarian cancer through a miR-335/DAAM1/myosin II axis-dependent mechanism
Source: J Ovarian Res. 2023 Jan 10;16:8. doi: 10.1186/s13048-022-01077-y (PMC9830800; doi:10.1186/s13048-022-01077-y)
Supplement: Supplementary file 2 — Additional file 2: Supplementary Table 1. Primer sequences for RT-qPCR. [file 13048_2022_1077_MOESM2_ESM.docx]

**Supplementary Table 1** Primer sequences for RT-qPCR

| Target | Sequence |
| --- | --- |
| FOXD3 | Forward 5’-GAAGCCGCCTTACTCGTACA-3’ |
|  | Reverse 5’-CGCTCAGGGTCAGCTTCTT-3’ |
| DAAM1 | Forward 5’-ACAGAGAAGCCATGTTTGCACT-3’ |
|  | Reverse 5’-AGAATTCAGGCCAACTTGTAGCTC-3’ |
| GAPDH | Forward 5’-ACATCATCCCTGCCTCTACTG-3’ |
|  | Reverse 5’-TTTGGCAGGTTTTTCTAGACGG-3’ |
| miR-335 | Forward 5’-TCAAGAGCAATAACGAAAAATGT-3’ |
|  | Reverse Reverse universal primer |
| U6 | Forward 5’-CTCGCTTCGGCAGCACATATACT-3’ |
|  | Reverse 5’-ACGCTTCACGAATTTGCGTGTC-3’ |
